# 3D Printing Today, AI Tomorrow: Rethinking Apert Syndrome Surgery in Low-Resource Settings

**DOI:** 10.3390/healthcare13151844

**Published:** 2025-07-29

**Authors:** Maria Bajwa, Mustafa Pasha, Zafar Bajwa

**Affiliations:** 1 MGH Institute of Health Professions (IHP), Boston, MA 02129-4557, USA; 2 REBEL Lab, MGH IHP Faculty, Boston, MA 02129-4557, USA; 3 College of Health Professions, Upstate Medical University, Syracuse, NY 13210-2375, USA; 4 CAE Consultants, Karachi 75500, Pakistan; mmupasha@gmail.com; 5 Department of Radiology, United Health Services Hospitals, Johnson City, NY 13790, USA; jaff1974@gmail.com; 6 Neurosciences and Radiology Clerkship, Binghamton Clinical Campus, Binghamton, NY 13790, USA; 7 The Norton School of Medicine, Upstate Medical University, Syracuse, NY 13210-2375, USA

**Keywords:** healthcare simulation, presurgical planning, surgical planning, apert syndrome, 3D printing, 3-dimensional printing, artificial intelligence (AI) and 3D printing, craniofacial surgery

## Abstract

**Background/Objectives:** This case study presents the first documented use of a low-cost, simulated, patient-specific three-dimensional (3D) printed model to support presurgical planning for an infant with Apert syndrome in a resource-limited setting. The primary objectives are to (1) demonstrate the value of 3D printing as a simulation tool for preoperative planning in low-resource environments and (2) identify opportunities for future AI-enhanced simulation models in craniofacial surgical planning. **Methods:** High-resolution CT data were segmented using InVesalius 3, with mesh refinement performed in ANSYS SpaceClaim (version 2021). The cranial model was fabricated using fused deposition modeling (FDM) on a Creality Ender-3 printer with Acrylonitrile Butadiene Styrene (ABS) filament. **Results:** The resulting 3D-printed simulated model enabled the surgical team to assess cranial anatomy, simulate incision placement, and rehearse osteotomies. These steps contributed to a reduction in operative time and fewer complications during surgery. **Conclusions:** This case demonstrates the value of accessible 3D printing as a simulation tool in surgical planning within low-resource settings. Building on this success, the study highlights potential points for AI integration, such as automated image segmentation and model reconstruction, to increase efficiency and scalability in future 3D-printed simulation models.

## 1. Introduction

Apert syndrome, also known as type I acrocephalosyndactyly, is a rare congenital disorder caused by missense mutations in the fibroblast growth factor receptor 2 (*FGFR2*) gene [1,2]. It is clinically defined by a triad of craniosynostosis (premature fusion of cranial sutures), midface hypoplasia (underdevelopment of the central facial region), and syndactyly (fusion of fingers, or mitten hands), which together result in a vertically elongated craniofacial profile and significant functional impairments in hearing, speech, and swallowing, and cognitive development later in life [3]. The syndrome affects approximately 1 in 60,000 to 88,000 live births [4,5], and although rare, it poses substantial clinical and surgical challenges, owing to the involvement of multiple systems with varying patterns in each individual [6].

Management of Apert syndrome necessitates a series of staged surgical interventions beginning in infancy [5]. Initial craniotomy is typically performed within the first year of life to address craniosynostosis, elevated intracranial pressure, and associated growth and cognitive problems later in life. Subsequent procedures include palate repair (around 6–14 months), syndactyly release (1–4 years), and midface or jaw advancement (4–6 years), depending on the individual’s growth and severity of anatomical anomalies [2]. Given the anatomical complexity and variability, detailed presurgical planning is essential for optimizing outcomes [7].

High-income settings increasingly use virtual surgical planning (VSP) tools, including interactive 3D visualization, 3D-printed models, and extended reality (XR) platforms, to enhance surgical accuracy, reduce intraoperative uncertainty, and lower surgeons’ cognitive load by providing them with a visual of the operative field [8,9,10,11]. In contrast, in many low-resource settings [12], surgical planning continues to rely on two-dimensional (2D) imaging, such as static CT scans and MRI images [personal communication with the surgical team [13,14], which lack spatial fidelity to adequately represent the intricate craniofacial anatomy [9]. As a result, surgeons encounter difficulties in visualizing and planning complex procedures, leading to increased surgical risk, prolonged operative times, and suboptimal outcomes as compared to 3D virtual imaging [15,16].

The limited adoption of advanced planning tools in low- and middle-income countries (LMICs) is primarily attributed to cost, infrastructure, restricted access to technology [10,17], as well as gaps in awareness, technical expertise, and clinical integration of biomedical engineering technologies. These challenges are pronounced in complex craniofacial conditions like Apert syndrome, which presents a broad spectrum of cranial deformities and anatomical variations across individuals. This clinical variability necessitates patient-specific planning strategies to ensure accurate, safe, and effective surgical outcomes [3,18]. Without access to patient-specific models, neurosurgical teams in LMICs often rely on conventional, less precise planning methods that are labor-intensive and prone to error [10]. Although basic 3D technology is becoming increasingly available over time, these limitations still present a critical gap in surgical planning capabilities, illustrating a need for affordable, scalable simulated planning solutions in low-resource settings.

To address this gap, this case demonstrates a low-cost, team-based application of 3D printing for presurgical planning in Apert syndrome, an approach that has not been previously documented in the literature. Notably, the project was initiated by the patient’s parents, a physician, and an engineer when they consulted the surgical team to explore planning options. The surgical team described relying on 2D CT scans, with critical decisions often made intraoperatively under pressure, likened to solving a “jigsaw puzzle” once the skull was opened. In response, the family proposed generating a 3D-printed model to aid planning. The surgical team accepted the proposal, and a model was co-developed for simulation in a non-urgent setting prior to the actual procedure. This early caregiver-led involvement is uncommon in surgical innovation and highlights how motivated families can catalyze clinical solutions in under-resourced settings. The aim of this report is to demonstrate the value of community-driven, context-appropriate collaboration for enhancing presurgical planning in low-resource settings and to propose a workflow for integrating artificial intelligence (AI) in future model reconstruction and automation.

## 2. Case Details

The patient was born at term via elective cesarean section following an unremarkable antenatal course. The indication for cesarean was adequate uterine contractions without fetal descent. No antenatal imaging suggested craniofacial or structural abnormalities. At birth, the neonate cried immediately and weighed 3.56 kg; however, formal APGAR scores were unavailable. Physical examination revealed features characteristic of Apert syndrome, including bilateral complex syndactyly of the hands and feet, brachycephaly with a flattened occiput, hypertelorism, midface hypoplasia, and a high-arched palate. The cranial morphology was suggestive of premature fusion of the coronal sutures. A clinical diagnosis of Apert syndrome was made at birth based on these findings. Initially, the infant exhibited normal tone and spontaneous activity but developed progressive respiratory distress within minutes. Nasal suctioning attempts were unsuccessful, and peripheral cyanosis ensued, prompting urgent transfer to the neonatal intensive care unit (NICU) and initiation of supplemental oxygen. In the NICU, the infant required nasal stenting and prolonged suctioning for persistent upper airway obstruction. Feeding difficulties were also prominent, contributing to early growth faltering.

Developmental delays were observed across multiple domains. According to caregiver reports, the infant appeared passive, showed minimal social engagement, did not smile responsively, and failed to react to voices or environmental sounds during the first few months. No babbling or vocalizations were noted by 4–5 months, indicating early delays in social, cognitive, and communicative development. Audiologic evaluation confirmed conductive hearing loss secondary to middle ear effusions, managed with tympanostomy tube placement.

## 3. Materials and Methods

Surgical planning for cranial vault remodeling began at nine months of age. The delay in surgery was due to a combination of factors, including the initial shock of an unanticipated complex diagnosis at birth, the newborn’s early respiratory and feeding complications, maternal recovery following cesarean section, and logistical delays related to planning and coordination, compounded by access-related challenges in an out-of-pocket healthcare system. The family contacted the surgical team before the child’s 9th month of age, and model development began shortly thereafter. Planning continued over the following months.

**Figure 1:**
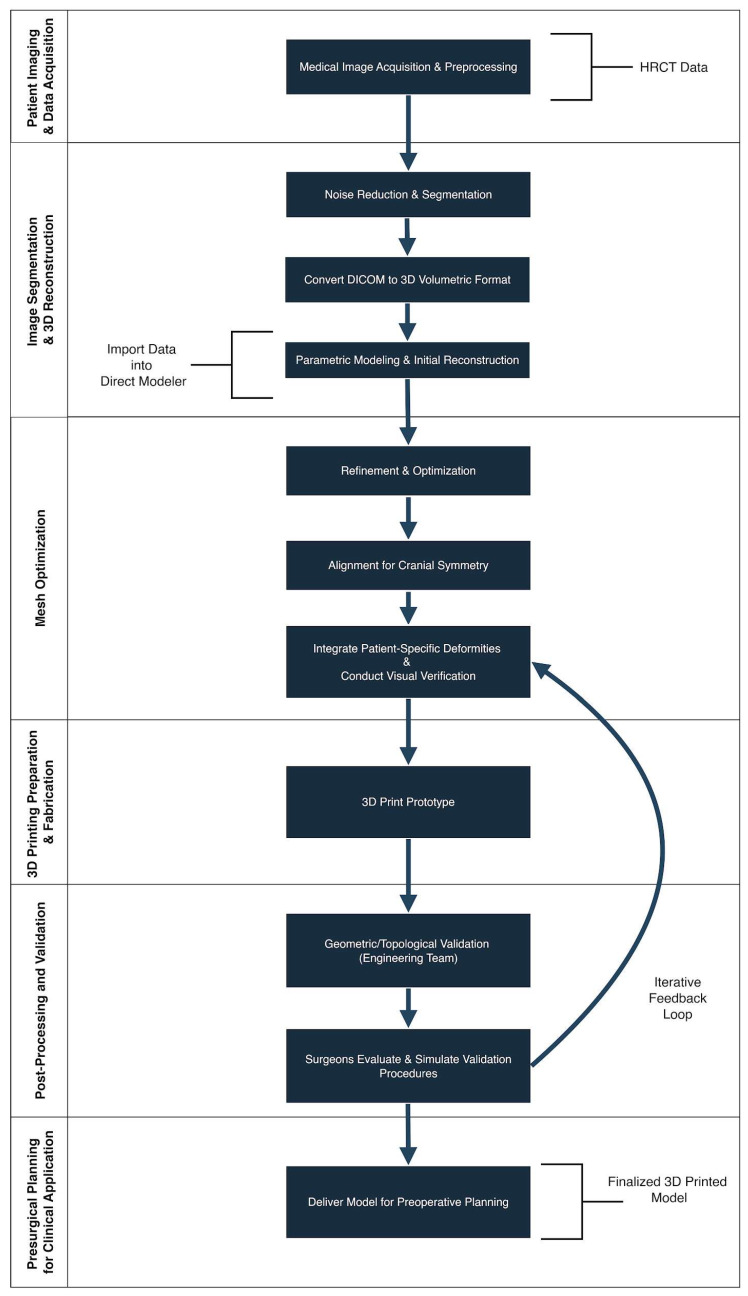
Workflow for the Reconstruction of a Patient-Specific 3D Cranial Model. Step-by-step diagram outlining the process from high-resolution CT data acquisition through segmentation, mesh generation, CAD editing, and 3D printing. This workflow was used to produce an accurate anatomical model of the infant’s cranium for pre-surgical planning in Apert syndrome.

This section outlines the step-by-step workflow for generating a patient-specific 3D-printed cranial model, which was subsequently used to simulate the patient’s cranial morphology and to plan the surgical approach. (Figure 1).

### 3.1. Patient Imaging and Data Acquisition

High-resolution computed tomography (HRCT) scans were acquired from a nine-month-old infant patient diagnosed with severe craniofacial malformations due to Apert syndrome. Axial images were obtained at slice intervals of 3.0 mm, providing adequate spatial resolution for anatomical reconstruction. All imaging data were stored in Digital Imaging and Communications in Medicine (DICOM) format for subsequent processing and analysis.

### 3.2. Image Segmentation and 3D Reconstruction

DICOM datasets were imported into InVesalius 3 [19], an open-source biomedical imaging platform. In HRCT imaging, each anatomical structure exhibits a distinct attenuation value based on tissue density, allowing visual differentiation. High-density tissues such as bone appear brighter due to higher attenuation, thus elevated Hounsfield Unit (HU) values [20]. Leveraging the HU values differences, initial segmentation was performed using a thresholding technique with an HU range of 1000 to 2000 to isolate specific structures from others. This technique effectively isolated cranial bone structures while excluding soft tissue, cerebrospinal fluid, and imaging artifacts. This HU range was selected based on established values for cortical and trabecular bone. Manual refinement tools were subsequently applied to enhance segmentation accuracy, particularly in areas affected by incomplete ossification or image noise.

Segmentation refinement was conducted using a combination of manual and semi-automated tools, including region-growing, contour editing, and slice-by-slice painting. Special attention was given to regions of incomplete ossification and prematurely fused sutures, which are characteristic of Apert syndrome. Non-cranial elements such as the mandible and vertebrae were manually excluded.

A detailed 3D surface mesh was created by converting the segmented volumetric data into a polygonal representation that accurately follows the contours of the cranial structures. This process involved identifying the boundaries between different tissue densities within the HRCT data and constructing a continuous surface delineating these regions. The resulting mesh, composed of interconnected triangular facets, captured the anatomical features with high fidelity. Once the surface reconstruction was complete, the mesh was exported in stereolithography (STL) format—a widely used file type in medical modeling and 3D printing—for further refinement and processing in computer-aided design (CAD) software, ANSYS SpaceClaim version 2021.

### 3.3. Segmentation Process Specifics

Each HRCT slice was individually reviewed to ensure accurate delineation of thin cranial bones and complex sutural morphology. Manual corrections were essential in areas with variable voxel intensities and imaging artifacts, particularly those introduced by neonatal restraint devices. The segmentation methodology was informed by prior work in pediatric craniofacial imaging [21], ensuring both anatomical fidelity and reproducibility.

### 3.4. Mesh Optimization Techniques

The initial STL mesh underwent geometric refinement to correct topological inconsistencies, such as non-manifold edges, holes, flipped normals, and self-intersections. These corrections were performed in ANSYS SpaceClaim [22], where additional smoothing, decimation, and sculpting were applied to enhance surface quality without compromising anatomical accuracy. The software enables facet-to-facet interaction within STL files, providing improved control for smoothing and refining the 3D model. Particular care was taken to preserve the morphology of cranial sutures, foramina, and other clinically relevant landmarks.

### 3.5. 3D Printing Preparation and Fabrication

The optimized STL model was imported into slicing software PrusaSlicer [23] to generate G-code for 3D printing. The model was fabricated using FDM on a Creality Ender-3 printer [24]. Printing parameters are summarized in Table 1.

Post-print, the model underwent acetone vapor smoothing to enhance surface finish and tactile clarity. The choice of materials and printing parameters was guided by the need to replicate the mechanical properties of neonatal cranial bones. Resin-based materials enabled high-resolution detailing, essential for depicting small anatomical structures, while offering variable rigidity: firm in areas requiring structural integrity and more pliable in regions simulating open sutures.

### 3.6. Post-Processing and Validation

**Figure 2:**
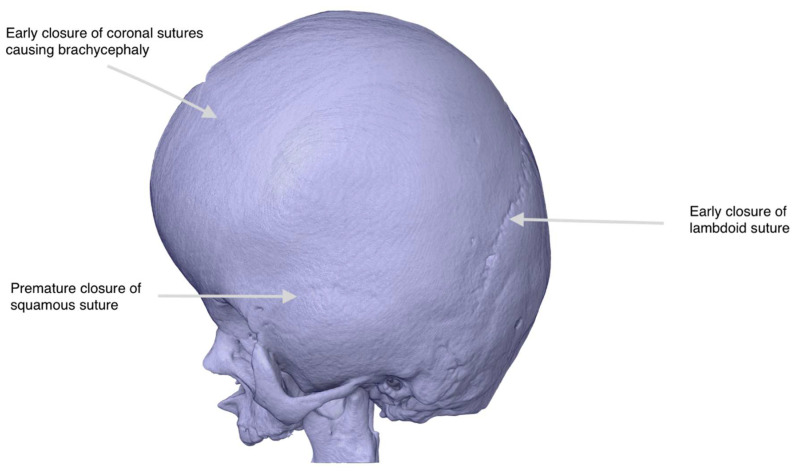
Digital Reconstruction of Cranial Anatomy for Preoperative Planning in Apert Syndrome. Patient-specific 3D CAD model generated from high-resolution CT data of an infant with Apert syndrome. The digital rendering highlights the hallmark features of craniosynostosis, including premature closure of the lambdoid, coronal, and squamous sutures, as indicated by arrows. The model was used for virtual surgical planning before fabrication.

After fabrication, the support structures were carefully removed, and the model underwent surface sanding, followed by optional painting to emphasize key anatomical features. Validation was conducted through visual inspection and dimensional comparison with the original HRCT data and the digital STL mesh, ensuring accurate representation of the patient’s cranial anatomy. The surgical team reviewed the model and provided feedback based on the HRCT, which was incorporated before finalizing the version used for surgical planning (Figure 2). The model was considered ready for use once the lead surgeon confirmed that it accurately reflected the surgical anatomy as visualized on the radiological scans and was sufficient for simulation and operative planning.

### 3.7. Presurgical Planning for Clinical Application

**Figure 3:**
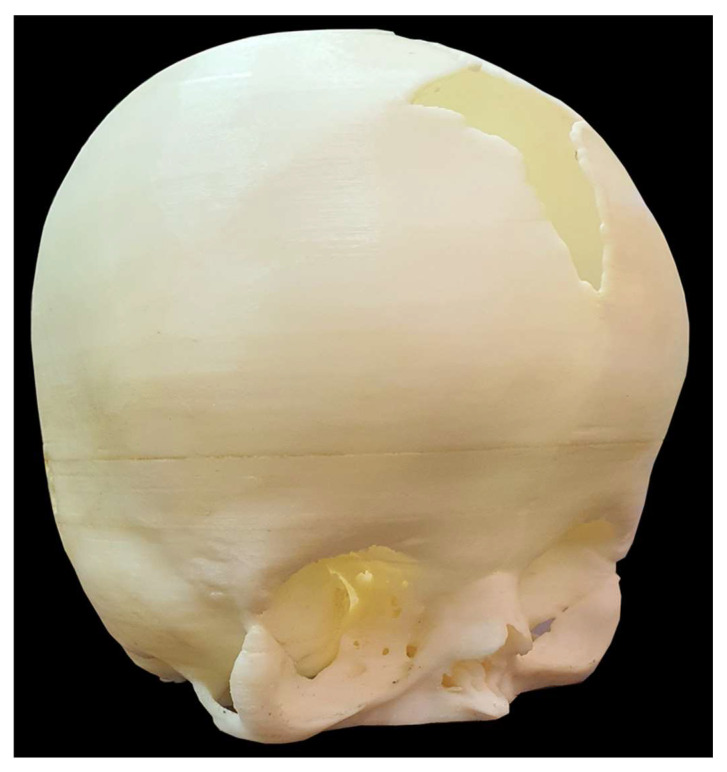
Physical 3D-Printed Cranial Model for Simulation and Surgical Planning. A tangible, full-scale cranial model fabricated from high-resolution CT data of an infant with Apert syndrome. This physical model, produced using FDM, was used for preoperative simulation, incision planning, and to improve spatial understanding of the patient’s cranial anatomy in a low-resource setting.

The multidisciplinary team of family caregivers, engineers, and surgeons convened on four separate occasions, ensuring thorough collaboration across multiple sessions. For a low-resource setting such as Pakistan, a novel approach of virtual surgical planning using 3D modeling and visualization was introduced to support preoperative planning. Surgeons utilized the model to assess surgical entry points, incision paths, and reshaping strategies, thereby reducing the risk of anticipated complications during surgery and improving the likelihood of optimal outcomes. The material properties of the model helped simulate cranial bone anatomy with tactile fidelity, allowing the surgical team to assess incision paths and anatomical relationships with greater confidence. The balance between the variable rigidity enabled the model to withstand repeated handling and better represent operative anatomy during planning, supporting more confident incision mapping and approach selection by the surgical team. After surgery, the model served as an educational tool for documenting procedural innovations and outcomes. Figure 3 shows an actual replica of the printed patient skull model that was provided to the surgeon for presurgical planning.

## 4. Results

The model was created from HRCT data acquired at 9 months using a segmented digital mesh and fabricated through the FDM, as detailed in the Methods. It was visually and dimensionally validated by the surgical team prior to use. The patient-specific 3D-printed cranial model was successfully used during presurgical planning to correct cranial deformities associated with Apert syndrome in this infant patient. Examining the inner curvature of the neonatal cranium enabled more precise presurgical mapping and incision planning. The surgical team utilized the model to simulate incision placement and visualize internal cranial structures, thereby facilitating the selection of surgical approaches tailored to the patient’s unique anatomy. Surgery was performed at 13 months of age and lasted approximately 10 h. The CT scan used for the 3D model was acquired at 9 months, and surgery took place at 13 months. Although cranial growth occurs in infancy, early suture fusion in Apert syndrome typically limits significant anatomical changes. The team maintained regular contact throughout, and the model’s accuracy was reaffirmed during the planning process. It remained valid for pre-surgical simulation.

Simulating multiple surgical approaches using the model reduced uncertainty during the procedure and helped mitigate potential complications. Due to the small operative field in neonates and the heightened risk of bleeding, having a physical reference was noted to assist with anticipating vascular challenges and managing intraoperative bleeding more effectively. While an iatrogenic durotomy did occur, the planned opening of the cranial sutures was executed successfully. The surgical team credited the simulated 3D-printed model with improving spatial orientation, enhancing decision-making, and reducing overall operative time by approximately four hours compared to similar cases performed without 3D model assistance.

**Figure 4:**
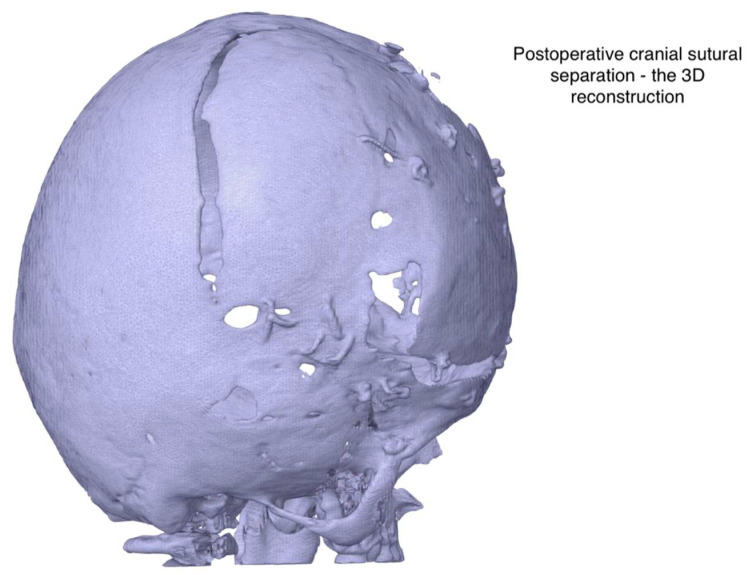
Postsurgical Digital Reconstruction Showing Cranial Remodeling. 3D visualization of the patient’s skull generated from postoperative imaging. The model illustrates surgical modifications, including suture release and bone remodeling, used to assess anatomical changes and postoperative alignment following cranial vault surgery for Apert syndrome.

The patient recovered postoperatively with no further complications. According to the surgical team, the simulation-based planning contributed to smoother intraoperative workflow and potentially aided the patient’s early postoperative recovery. Postoperatively, functional improvements were noted, including increased mobility, communication, and social engagement. Figure 4 shows post-surgical 3D models used to assess recovery and anatomical development over time.

## 5. Discussion

This study demonstrates that a low-cost, patient-specific 3D-printed cranial model supported simulated presurgical planning for a pediatric Apert syndrome case in a low-resource setting. The following discussion examines the technical feasibility, implementation context, system-level challenges, and clinical outcomes associated with using 3D-printed models for presurgical planning in low-resource settings.

### 5.1. Feasibility and Accessibility of 3D Planning in Low-Resource Settings

The modeling pipeline was built entirely with low-cost, consumer-grade technology and open-source or commercially accessible software. A Creality Ender-3 printer [24] and ABS filament were used to fabricate the model, and the entire process was completed for $40, which included labor, delivery, and printing wages. The low cost demonstrates the cost-effectiveness and potential for widespread accessibility of commonly used 3D printing technology and methodology. (See more details in Supplementary Materials S1). This is a negligible expense compared to the average total cost of a craniotomy in Pakistan, which can be as high as $4500. Segmentation was completed in InVesalius 3, an open-source medical imaging platform [19], and mesh refinement in ANSYS SpaceClaim [22]. The segmentation, modeling, and CAD preparation required approximately 5 h, while 3D printing took an additional 6 h. This setup demonstrates that meaningful presurgical planning tools can be developed without high-end surgical simulation platforms, provided there is sufficient technical and pedagogical expertise. While the process requires time and specialized skills, its affordability and adaptability make it viable for use in resource-constrained healthcare environments. Beyond feasibility, the model had direct intraoperative value, helping mitigate uncertainty in a high-risk pediatric case.

### 5.2. Health System Challenges: Infrastructure and Expertise

Despite the growing availability of 3D printing tools, their integration into surgical workflows remains limited in many health systems due to gaps in infrastructure, funding, and training [13]. This case highlights how surgical teams often lack access not only to equipment but also to trained personnel capable of bridging the technological gap [10]. Addressing this gap requires more than technical solutions; it calls for interprofessional systemic support and investment to enable simulation-based planning as a standard of care, especially in high-stakes pediatric craniofacial surgeries [16]. In many low-resource settings, healthcare data are often not captured and not stored in standardized, retrievable digital formats, contrary to the norm in relatively resource-rich countries. Even when digitized, the computational capacity needed for advanced AI-driven analysis remains largely inaccessible, resulting in difficult integration and posing a major barrier to AI adoption [25].

### 5.3. Local Innovation and Stakeholder-Driven Implementation

The workflow in this case was developed and executed by an independent engineering team, in collaboration with the patient’s family and the surgical team, rather than through institutional or clinical channels. This highlights the potential of decentralized innovation in low-resource settings, where solutions are often driven by motivated individuals outside the formal healthcare system in the presence of open-minded, solution-focused care providers. It also highlights the active role that caregivers can play in initiating care-enhancing interventions when supported with technical resources and clinical openness. Recognizing and supporting such models could help expand patient-centered innovation globally, a concept supported by a private-public partnership, as evidenced in the literature [10]. Importantly, this caregiver-informed, low-cost planning workflow may be adapted beyond Apert syndrome to address other complex congenital anomalies requiring anatomical simulation. Its reliance on accessible tools, non-institutional collaboration, and iterative feedback demonstrates a scalable model for surgical innovation in similarly constrained environments.

### 5.4. Clinical Insights

This case illustrated the feasibility of adopting simulation-based presurgical planning in a setting where such practices are not routine. By creating a patient-specific 3D model, the surgical team was able to visualize anatomical complexity and simulate osteotomy planning before entering the operating room. This simulated rehearsal improved spatial orientation and incision accuracy, contributing to an estimated four-hour reduction in operative time as reported by the surgical team, which is supported by the literature [16]. Simulating osteotomies and visualizing complex anatomy preoperatively enabled safer execution in a high-risk neonatal case with a limited operative field and vascular fragility. These findings align with previous reports that patient-specific 3D-printed models improve spatial orientation and reduce intraoperative uncertainty in craniosynostosis surgeries [16].

While this study focused on Apert syndrome, 3D printing has also been explored and successfully applied in several other congenital syndromes with craniofacial deformities to aid presurgical planning [16]. Syndromes, such as Pierre-Robin, Treacher Collins, Pfeiffer, Crouzon, and others, each with their characteristics, genetically driven craniofacial anatomical defects, have shown improved preoperative surgical planning, functional outcomes, and aesthetics when 3D models were used [8].

## 6. Innovation Outlook: Future Applications and Technologies

### 6.1. Proposed AI Applications in the Surgical Planning Workflow

Integrating AI into neonatal skull model reconstruction represents a significant advancement in medical imaging and surgical planning. By automating complex tasks, improving accuracy, and offering predictive capabilities, AI effectively addresses many limitations of traditional workflows [26]. To maximize its clinical impact, future efforts should focus on developing and training AI algorithms tailored to a wide range of craniofacial conditions, supported by comprehensive anatomical datasets [27]. Interdisciplinary collaboration among clinicians, AI researchers, and engineers will be vital in translating these innovations into real-world healthcare solutions.

The current workflow relies heavily on manual segmentation and model refinement. To address limitations in accuracy and labor intensity, we propose integrating artificial intelligence (AI) in two key domains: image segmentation and 3D reconstruction.

#### 6.1.1. AI-Enhanced Image Segmentation

Deep learning models, particularly convolutional neural networks (CNNs), such as U-Net and attention-based neural networks (CA-Net), offer potential for automating and enhancing cranial bone segmentation [28]. These models can learn hierarchical features from training data, overcoming challenges posed by variable Hounsfield values, incomplete ossification, and imaging noise [29]. Attention mechanisms in CA-Net could further improve precision by focusing on fused sutures and complex bone morphologies characteristic of Apert syndrome.

#### 6.1.2. AI-Driven 3D Reconstruction

AI techniques such as Principal Component Analysis (PCA) can minimize alignment errors by analyzing geometric features and anatomical landmarks, optimizing slice alignment, and ensuring a smooth transition between layers [30]. In cases of incomplete imaging or missing data, Generative Adversarial Networks (GANs) can predict and fill in gaps by learning from the surrounding data, ensuring that the reconstructed 3D model remains continuous and anatomically realistic [31]. AI-assisted computer-aided design (CAD) tools could further automate mesh healing, artifact removal, and smoothing processes. Generative AI, such as CA-Net and GAN, could streamline the current workflow, reduce human error, and enable faster production of high-fidelity models suitable for surgical planning [29]. See Table 2.

#### 6.1.3. Barriers to AI Adoption

Despite the demonstrated feasibility of using low-cost, open-source tools to reconstruct and 3D print a neonatal cranium affected by Apert syndrome, several systemic barriers continue to hinder the broader adoption of such AI-assisted workflows in LMICs. One significant limitation is the restricted resolution of available HRCT data, which often lacks the fine anatomical detail necessary for accurate craniofacial reconstruction, particularly in neonates where bone sutures and fontanelles are still developing. Additionally, data scarcity, especially in terms of annotated pediatric craniofacial datasets, limits the applicability of machine learning models for automated segmentation and classification. Computational infrastructure also poses a significant barrier. Many institutions in LMICs lack access to high-performance hardware or cloud-based platforms needed for processing large volumetric datasets or training deep learning algorithms. In our case, the segmentation process alone required over 5 h, largely due to the lack of advanced computational infrastructure, such as Graphics Processing Unit (GPU)-accelerated processing or cloud-based segmentation platforms. Furthermore, technical expertise in biomedical modeling, AI integration, and post-processing remains unevenly distributed. These challenges, coupled with regulatory gaps and resource constraints, underscore the need for context-sensitive innovation and collaborative capacity building to ensure that AI-enabled surgical planning tools become accessible and impactful across under-resourced healthcare systems.

### 6.2. Exploring Extended Reality for Preoperative Simulation and Planning

**Figure 5:**
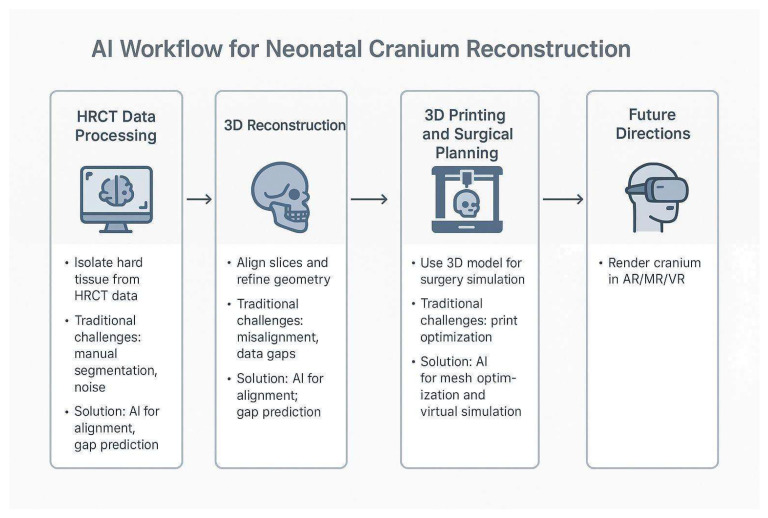
Conceptual AI-Integrated Workflow for Neonatal Cranial Reconstruction. The diagram illustrates a proposed workflow for 3D visualization and planning in neonatal cranial surgery. The process includes: (1) HRCT data processing, (2) 3D reconstruction, (3) 3D printing, and surgical planning. A final node, “Future Directions,” highlights opportunities for artificial intelligence to automate segmentation, enhance model accuracy, and improve clinical integration in resource-constrained environments.

A compelling future direction for this work involves extending the 3D cranial model into immersive environments, such as extended realities, including virtual reality (VR), augmented reality (AR), or mixed reality (MR). These technologies would allow interactive exploration of patient-specific anatomy, enhancing medical education, preoperative planning, and patient communication [32]. VR simulations could offer fully immersive manipulation of cranial structures, while AR and MR could enable real-time overlay of the model onto the patient for intraoperative guidance [32]. Such systems could also support remote collaboration among specialists, improving diagnostic accuracy and treatment planning. Future research could focus on developing a seamless integration between 3D reconstruction pipelines and real-time rendering engines to ensure high-fidelity visualization in clinical and research contexts. Figure 5 illustrates the integration of AI into the workflow for 3D visualization in neonatal cranial reconstruction.

## 7. Strengths and Limitations

This study demonstrates several key strengths. It highlights a caregiver-initiated, interdisciplinary collaboration between the patient’s family and the engineering and surgical teams, which led to the successful creation of a patient-specific planning model without formal institutional support or a standard of care. The surgical team was receptive and integrated the model into their planning process, reflecting open-minded clinical engagement. The workflow used accessible tools and low-cost materials, making it replicable in similar low-resource contexts. The case also reinforces the potential for stakeholder-driven innovation to address surgical planning gaps, especially in pediatric craniofacial care.

While several strengths were exhibited, the limitations were also present. The engineering team validated the model against the HRCT data, while the surgical team performed a visual assessment but did not quantitatively compare the model to the original imaging. This study reports a single case and, therefore, the workflow’s generalizability remains untested. Manual segmentation and mesh processing remain time-intensive, requiring anatomical knowledge and CAD proficiency, indicating the need for future development of automated segmentation pipelines, accuracy benchmarking, and multi-case evaluations. Despite these constraints, the case supports the value of accessible, team-driven modeling workflows in under-resourced surgical contexts.

## 8. Conclusions

This case demonstrates that low-cost, patient-specific 3D-printed models can facilitate detailed surgical planning and mitigate intraoperative uncertainty in Apert syndrome, even in low-resource settings. Despite one postoperative complication, the approach led to a markedly shorter operative time, approximately four hours, which is associated with faster healing and fewer complications compared to similar cases. While the outcome demonstrates a clear benefit, it reflects a single case experience. A broader evaluation across multiple cases is needed to determine reproducibility, clinical impact, and feasibility at scale. To address current limitations in accuracy, efficiency, and scalability, future work should explore the integration of artificial intelligence (AI), particularly in automating image segmentation and enhancing 3D reconstruction. Leveraging AI in these domains may streamline model generation, reduce operator dependency, and enable wider adoption in low-resource surgical settings. Multicenter trials are warranted to systematically evaluate the effectiveness of both AI-assisted and non-AI-based preoperative planning approaches for complex craniofacial conditions across diverse clinical settings. In our case, the use of open-source tools was crucial in making this intervention feasible in a resource-constrained environment. To support similar innovations globally, collaborative open-source platforms should be encouraged to enable knowledge sharing, validation, and broader clinical adoption.

## Figures and Tables

**Table 1:** 3D Printing Parameters for Neonatal Cranium Model Fabrication.

Parameter	Specification
Material	Acrylonitrile Butadiene Styrene (ABS)
Nozzle Diameter	0.4 mm
Nozzle Temperature	230 °C
Bed Temperature	100 °C
Layer Height (Resolution)	0.1–0.2 mm
Infill Density	10–20% (solid infill used for structural strength)
Print Scale	1:1 anatomical accuracy
Structural Supports	Enabled for overhanging structures
Total 3D Printing Costs (including Labor)	$40

**Table 2:** Comparison of the 3D Printing Workflow with and without AI.

Aspect	Traditional Workflow	AI-Assisted Workflow
Workflow Stages	HRCT data acquisition manually calibrated for neonatal imaging. Manual segmentation using HU thresholds for bone isolation. Manual slice alignment using anatomical landmarks. 3D model generation through stacking and interpolation algorithms. Physical prototyping via STL file generation and 3D printing.	Automated preprocessing using AI-based denoising and contrast adjustment. Deep learning segmentation with CNNs like U-Net for high-precision extraction of cranial structures. AI-enhanced alignment using PCA and landmark detection to reduce registration errors. 3D reconstruction using GANs or parametric models for continuity. Simulation & integration with AR/VR platforms for surgical rehearsal.
Challenges	- Subjectivity in thresholding HU values leads to inconsistent bone isolation. - High manual workload across multiple stages. - Misalignment due to variability in slice capture and anatomical complexity. - Inconsistencies in model resolution and anatomical fidelity. - No built-in prediction or simulation capability for post-reconstruction analysis.	- Limited pediatric data makes model training difficult. - Variability in skull morphology across patients hinders generalization. - Imaging noise and artifacts impair segmentation clarity. - Incomplete ossification in neonates leads to gaps in reconstructed models. - Validation of synthetic AI-generated models remains a regulatory challenge.
Proposed Solutions	- Expert-based corrections post-thresholding. - Multiple segmentation passes with manual refinements. - Radiologist supervision for anatomical alignment and accuracy. - Post-processing tools for mesh cleanup (e.g., smoothing, gap filling).	- Data augmentation to synthetically expand neonatal datasets. - Transfer learning using adult cranial datasets as pretraining. - GANs and VAEs to reconstruct missing cranial regions. - Principal Component Analysis (PCA) for automated and accurate slice registration. - Attention mechanisms in CA-Net to improve focus on critical features like fused sutures.
Model Output	- Basic anatomical models with moderate fidelity, suitable for rough planning. - Inconsistent geometry due to manual interpolation and alignment. - STL files often require extensive healing before printing.	- High-resolution, patient-specific 3D reconstructions with smooth surfaces and anatomical accuracy. - Predictive simulations of cranial growth and suture development over time. - Ready-to-print STL/VR-compatible files generated with minimal manual intervention.
Clinical Integration	- Primarily used for visual inspection and orientation by the surgical team. - Rarely used for simulation due to time and effort involved. - Limited in use for interdisciplinary communication or educational engagement.	- Customized surgical planning with quantitative anatomical analysis. - Real-time intraoperative navigation using AI-generated models. - Seamless integration with virtual and mixed reality platforms for enhanced visualization and collaboration across departments.
Impact on Practice	- Time-intensive process, often taking several days from scan to print. - High variability between technicians affects model quality. - Scalability issues due to human resource dependency. - Limited reproducibility and objective validation.	- Significant time reduction—from hours to minutes in some stages. - Reduced inter-observer variability due to standardized AI protocols. - Enhanced model accuracy with detailed, realistic bone representation. - Scalable and reproducible workflow adaptable to other craniofacial conditions.

## Data Availability

Data is contained within the article or supplementary material.

## Supplementary Materials

The following supporting information can be downloaded at: https://www.mdpi.com/article/10.3390/healthcare13151844/s1. Refs. [33,34,35,36,37] are cited in the Supplementary Material file.
