# Supplementary material for: 3D Printing Today, AI Tomorrow: Rethinking Apert Syndrome Surgery in Low-Resource Settings
Source: Healthcare (Basel). 2025 Jul 29;13(15):1844. doi: 10.3390/healthcare13151844 (PMC12346539; doi:10.3390/healthcare13151844)
Supplement: Supplementary file 1 [file healthcare-13-01844-s001.zip › healthcare-3704074-supplementary.pdf]

Supplementary Material S1:

HRCT Data Processing of Cranial Tissue

High-resolution computed tomography (HRCT) scans were acquired from a ninth-month-old infant patient diagnosed with Apert syndrome. HRCT scans were obtained using imaging equipment with a precise slice thickness (0.6 mm – 3.0 mm), capturing fine anatomical details. The imaging data revealed various density gradients corresponding to different tissue types and materials of constraint. The skull, made of hard tissue, showed high attenuation values, appearing as the brightest area in the images. Traditional segmentation methods were used to isolate cranial bone structures, requiring meticulous manual analysis of each slice. The segmentation process focused on isolating cranial bone from surrounding tissues, such as the brain and soft tissues, through a combination of thresholding and manual refinement techniques. Specifically, thresholding involved selecting a specific range of Hounsfield units (HU) associated with bone tissue, typically between 1000 and 2000 HU *(Hounsfield, 1979)*. This approach, widely regarded as one of the most straightforward methods for segmenting cranial models from 3D radiographic volumes, is complemented by manual segmentation to refine cranial bone boundaries and correct inaccuracies (Didziokas, 2024).

The quality of the scans is influenced by the type of scanning equipment used, the duration of the examination, tube voltage and current, spatial voxel dimensions (radiographic densities), the field of view, and the characteristics of the object being scanned. HRCT often generates varying voxel intensities for similar structures across different regions of the scanned volume. As a result, determining an accurate threshold value for bone segmentation is a crucial yet challenging step, especially in manually performed procedures *(Molteni et al. 2013)*.

Despite the advancements in HRCT imaging, traditional processing methods face challenges, especially in managing large data volumes and ensuring accuracy.

3D Reconstruction and Computer-Aided Design (CAD)

After segmentation, the 2D slices were combined into a 3D model using a stacking algorithm. Any misalignments between slices were corrected by referencing anatomical landmarks and manually adjusting. Gaps in data, often due to incomplete imaging, were filled using interpolation techniques. The initial 3D model underwent further refinement to enhance its geometry. This involved removing artifacts and smoothing surfaces to create a realistic and anatomically accurate representation. The resulting model served as a digital foundation for physical modeling. Maintaining fidelity to the patient's unique cranial anatomy was crucial during reconstruction. Such details needed to be preserved from the high-resolution medical images to the reconstructed 3D model. Figure S1 captures the minute intricacies of the cranial foramina.

Figure S1: Illustrates cranial foramina

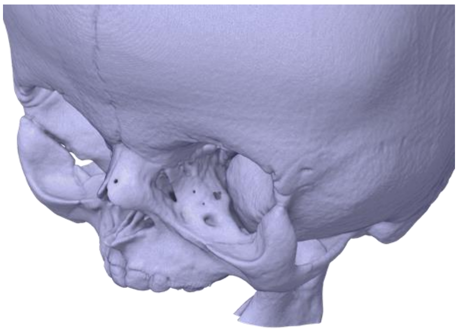

Each slice was carefully reviewed to identify features such as premature sutural fusions (Figure S2), characteristic of Apert syndrome. This laborious process involved highlighting areas of interest and correcting inconsistencies, ensuring the anatomical accuracy of the reconstructed model.

Figure S2: Premature sutural fusion

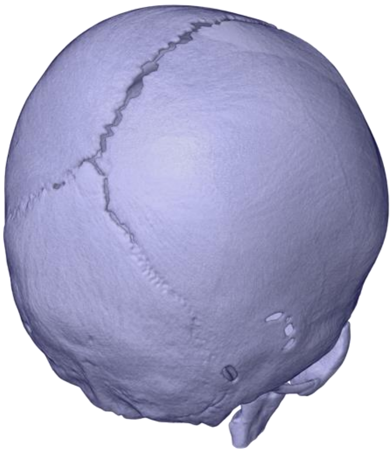

Refinement, healing, and adjustment of the 3D model were augmented using parametric modeling techniques. Parametric modeling involves using mathematical equations to define the geometry of the model, allowing for precise control over the shape and dimensions of the model. The refinement process included smoothing the model's surface from tessellated faces, correcting any geometric inaccuracies, and ensuring that the model accurately represents the patient's unique cranial anatomy.

Direct modeling software is widely used for processing, modifying, and refining 3D models, making it particularly effective for handling cranium reconstructions generated from medical imaging data. The software offers an intuitive toolset that enables users to make geometric adjustments such as realignment, scaling, rotation, and position adjustments to match anatomical references. Its direct editing tools allow for precise modifications to cranial sutures or bone structures, addressing variations due to growth, deformation, or pathology. The direct modeler can optimize surface features by refining smoothness and curvatures after importing the file, usually in the STL file format from the medical imaging software. This is particularly important for tessellated surfaces commonly encountered in 3D reconstructed models derived from medical

imaging. Cleaning tools were used to remove extraneous artifacts, smooth rough edges, and refine the mesh by adjusting polygon density, balancing model accuracy with computational efficiency for downstream applications. Additionally, the repair features abridged gaps, merged disconnected surfaces, and patched holes, ensuring the model remained anatomically intact. At that stage, the 3D reconstructed and refined cranium was ready for interactive study or manipulation in virtual space, making it suitable for rapid prototyping. Figure 3 illustrates the finalized 3D model of the neonatal cranium, now prepared for 3D printing.

Manually reconstructing a neonatal 3D cranium from an STL file was a labor-intensive and error-prone process that demanded significant experience and time. The challenge began with handling the artifacts, irregularities, and missing data typically encountered in models generated from medical imaging. The need to meticulously manipulate the 3D model by making geometric adjustments—realigning, scaling, rotating, and positioning the cranium—required extensive expertise to ensure accurate anatomical representation, showcased in Figures S3-S6.

Figures S3 through S6 show different views of the skull.

Figure S3 here.

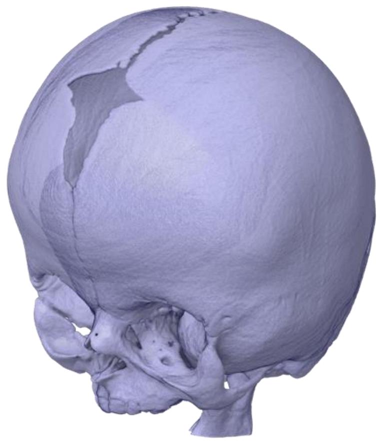

Figure S4 here.

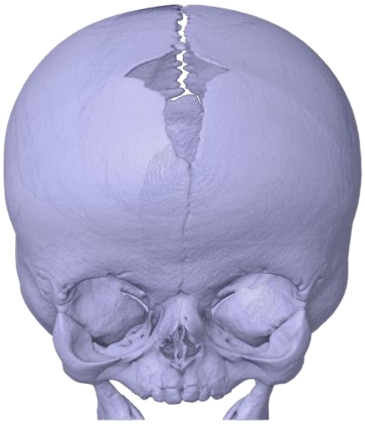

Figure S5 here.

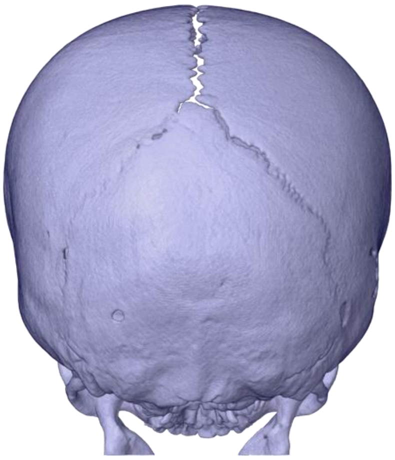

Figure S6 here.

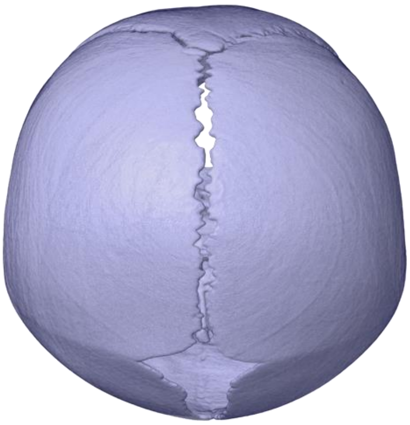

Furthermore, manually addressing variations due to growth, deformation, or pathology in cranial sutures or bone structures introduced another layer of complexity. The left and right lateral views, Figures S7 & S8, effectively highlight the distinctive cranial topology characteristic of Apert syndrome patients.

Figure S7 here.

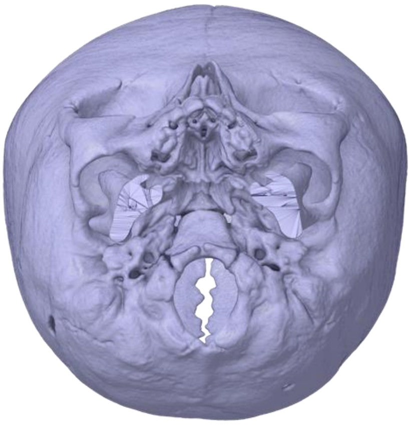

Figure S8 here.

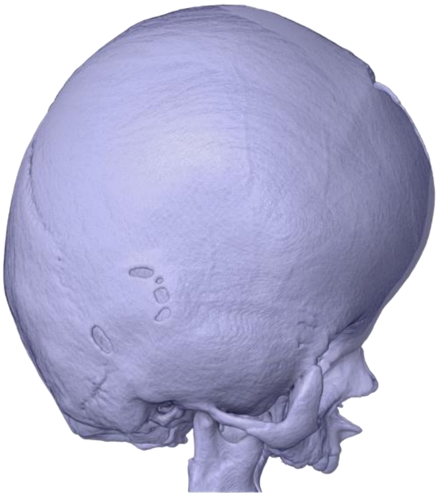

Optimizing surface features, such as refining smoothness and curvatures, involved time-consuming trial-and-error to achieve the desired result. Cleaning and repairing the model proved to be both time-consuming and computationally intensive, requiring significant effort and processing resources. Repairing the model, including bridging gaps, merging disconnected surfaces, and patching holes, was painstakingly performed and required great attention to detail to maintain anatomical integrity. Automating and streamlining these processes was essential to enhancing the accuracy and overall precision of anatomical models.

3D Printing

The finalized digital model was fabricated into a physical prototype using Fused Deposition Modeling (FDM), a widely adopted 3D printing technique recognized for its cost-effectiveness and broad material availability. FDM was chosen for its ability to quickly produce durable prototypes, utilizing thermoplastic filaments such as Acrylonitrile Butadiene Styrene (ABS) in this case. This material is well-suited for functional

testing and structural evaluation. This technology excels in creating functional models that are mechanically robust and suitable for fit, form, and basic functional testing. While FDM may not match the ultra-fine detail of resin-based processes, it offers reliable performance for structural prototypes and design validation. Its layer-by-layer extrusion approach enables the construction of complex geometries with reasonable accuracy and strength, making it a practical choice for early-stage product development. The choice of material and fabrication technique was made due to its durability, lightweight nature, and ease of 3D printing. The goal was to have an accurate, durable anatomical model to aid in pre-operative planning and procedure. The affordability and speed of FDM printing facilitated multiple design iterations, supporting an agile development cycle while minimizing production costs and lead times. The final 3D-printed model, shown in Figure 3, served as a surgical aid during preoperative planning, providing valuable anatomical insight to support clinical decision-making.

Supplementary Material S2:

Advanced AI algorithms, particularly convolutional neural networks (CNNs), have revolutionized the segmentation of high-resolution computed tomography (HRCT) images by automating the extraction of high-density structures such as cranial bones. Traditional segmentation methods rely heavily on manual thresholding techniques, which are often inconsistent due to varying voxel intensities and imaging conditions. In contrast, deep learning models like CNNs learn hierarchical spatial features from large datasets, allowing them to accurately differentiate cranial structures without manual input. These models significantly reduce human error and effort while maintaining segmentation accuracy exceeding 95% (Litjens et al., 2017), thereby improving efficiency, consistency, and reproducibility in medical imaging workflows.

Among these models, the U-Net architecture has been widely adopted for biomedical image segmentation due to its ability to preserve spatial detail and perform pixel-wise classification. However, it faces limitations when dealing with image artifacts, low contrast, and incomplete data, particularly in neonatal HRCT scans, where bones may not be fully ossified. These challenges are effectively addressed by advanced architectures such as the Comprehensive Attention-based CNN (CA-Net), which leverages spatial and channel-wise attention mechanisms to enhance focus on critical anatomical features, including prematurely fused sutures in conditions like Apert syndrome (Gu et al., 2021). CA-Net's adaptive capability makes it more robust in handling anatomical variations and incomplete or noisy data, thereby advancing the precision and applicability of AI-driven segmentation in clinical contexts.

Following segmentation, the 2D HRCT slices are compiled into a 3D model using stacking algorithms and exported as STL files for further processing in CAD tools. This 3D reconstruction process is traditionally manual and prone to error, involving artifact removal, surface smoothing, and anatomical corrections. AI models, including CNNs and CA-Net, can be further trained to automate aspects of this reconstruction, such as artifact elimination, smoothing of rough surfaces, and mesh optimization. While full automation is not yet achievable, AI significantly reduces manual workload and enhances the anatomical accuracy of the final 3D model, making it highly suitable for advanced applications like finite element analysis, surgical planning, and patient-specific medical interventions.

References

1. Hounsfield G. Computed Medical Imaging. Nobel Lecture, December 8, 1979. J Comput Assist Tomogr. 1980;4(5):665-74. doi:10.1097/00004728-198010000-00017 – Pubmed.
2. Didziokas, M., Pauws, E., Kölby, L., Khonsari, R.H. & Moazen, M. (2024) BounTI (boundary-preserving threshold iteration): A user-friendly tool for automatic hard tissue segmentation. Journal of Anatomy, 245, 829–841. https://doi.org/10.1111/joa.14063
3. Molteni R. Prospects and challenges of rendering tissue density in Hounsfield units for cone beam computed tomography. Oral Surg. Oral Med. Oral Pathol. Oral Radiol. 2013;116:105–119. doi: 10.1016/j.oooo.2013.04.013.
4. Litjens, G., Kooi, T., Bejnordi, B. E., Setio, A. A. A., Ciompi, F., Ghafoorian, M., van der Laak, J. A. W. M., van Ginneken, B. & Sánchez, C. I. (2017). A survey on deep learning in medical image analysis. Medical Image Analysis, 42, pp. 60–88.

5. Gu R, Wang G, Song T, Huang R, Aertsen M, Deprest J, Ourselin S, Vercauteren T, Zhang S. CA-Net: Comprehensive Attention Convolutional Neural Networks for Explainable Medical Image Segmentation. IEEE Trans Med Imaging. 2021 Feb;40(2):699-711.
